# Tennessee State Medical Society

**Published:** 1890-05

**Authors:** 


### TENNESSEE STATE MEDICAL SOCIETY.

---

FIFTY-SEVENTH ANNUAL MEETING HELD IN MEMPHIS, TENNESSEE, APRIL 8TH AND 9TH, 1890.

---

### APRIL 8TH—FIRST DAY—MORNING SESSION.

The Society met in the Ladies' Ordinary of the Gayoso Hotel, and was called to order by the President, Dr. Duncan Eve, of Nashville, at 10 a. m.

The Address of Welcome was delivered by Dr. D. D. Saunders, of Memphis.

The first paper was read by Dr. A. J. Swaney, of Gallatin entitled

### INCONTINENCE OF URINE IN CHILDREN.

He said the state of the bladder and the relative development and activity of the spinal cord and brain must be principally considered first, for the bladder in infants and young children there is a relatively powerful detrusor and feeble sphincter. The sphincter muscle that guards the entrance of the bladder has not sufficient resistance in many cases to properly resist the action of the vigorous detrusor.

The various causes he tabulated as follows: (1) Excessive acidity of the urine, which is usually caused by excessive formation of uric acid. (2) Over irritability of the muscular coat of the bladder, even when the urine is neutral. (3) Weakness of the sphincter. In such a case the urine is not passed rapidly or in a full stream, as when the cause is in a forcibly acting detrusor. (4) From reflex action, any source of irritation in or about the genital tract may be a cause of incontinence of urine. Local examination may discover a balanitis or adhesions between the prepuce and glans penis, with smegma around the corona. Two such cases had come under his observation in the last year. (5) Too great a quantity of urine, from the child drinking too large a quantity of fluids. (6) Vesical Calculi. (7) Malformation of the bladder. Several causes have been reported where persistent and irremediable incontinence was induced by one or both ureters opening into the urethra. (8) Contraction of the walls of the bladder, owing to hypertrophy of the muscular layer reducing the capacity of the bladder to one or two ounces. This he found to be the condition of the bladder in a case which he would report. The walls of the bladder were contracted so much that the patient's bladder would not hold more than one and a half ounces of water.

Treatment must depend upon the discovery of the proper cause in each specific case. Where the urine is highly acid it should be neutralized by the administration of alkalies. The acetate of potash is one of the best preparations for this purpose. Bicarbonate of potash or bicarbonate of soda may be used for the same purpose. Fruits often do well in children old enongh to partake of them, as the vegetable acids are changed to alkaline bases in the system. Where the fault is from the detrusor acting too vigorously, belladonna serves as a specific. Five drops of the tincture of belladonna may be given three times a day at first and the dose increased by single drops until there is some dryness of the throat and flushing of the skin. When the drug produces these effects it will control the incontinence if such effects are to be produced. For a lack of force in the sphincter ergot in full doses is indicated. A good effect follows the injection of five or six drops of the fluid extract of ergot into the connective tissue of the ischio-rectal fossa, as recommended by Dr. H. D. Chapin, of New York. In all cases of incontinence of urine everything possible should be done to tone up the nervous system, and to this end the essayist speaks of syrup iodide of iron and strychnine in full doses as being very valuable.

Dr. T. J. Happel, of Trenton, contributed a paper entitled a "Résume of Surgical Cases in a Country Physician's Practice," in which he outlined his general method of procedure at a considerable length, and said that the nearest approach to what might be termed "ideal surgery" that it had been his lot to deal with was one of his latest operations—the removal of what appeared to be a cancerous degeneration of a naevus or mole in an old lady, nearly 93 years of age. This tumor had attained to the size of a duck-egg, was growing on the left temple, and had developed rapidly within less than six months. It was removed under chloroform on April 21, 1889, and the patient was dismissed cured on May 5th. There was no elevation of temperature, and less than one quarter teaspoonful of pus.

In all his operations he uses chloroform. Theoretically, ether is safer, but much depends upon the manner in which the anæsthetic is used. Both are unsafe in careless hands, chloroform being much more so than ether, but when the respiration and pulse are carefully watched he considers chloroform as safe as ether. He finds that anæsthesia is much more rapidly produced by chloroform, and much less nausea following its use. As to mixing anæsthetics, he had no experience and less faith, as, owing to the different rates of volatilization of the ingredients, the patient uses but one at a time in reality.

Dr. Happel summarized his surgical work as follows: Six amputations of the lower extremities; five amputations of upper extremities; seven amputations of fingers and toes; two resections; five operations for caries or necrosis of the long bones; four cases of perineorrhaphy; one case of trachelorrhaphy; two of epithelioma of the lower lip; two for hare-lip; one of stone in the bladder; one of epulis; a large number of cases of fistula in ano; and many cases of rectal ulcers and internal hemorrhoids, with recovery in every case except one case of tubercular ulceration of the rectum.

He has twice amputated a mammary gland foı cirrhus, with recovery in both cases from the operation; but death in one case from recurrence in less than six months, and the other within less than two years. A similar result obtained in the encephaloid cancer. The fatal case of synovitis of the knee joint could not at the time he saw it have been expected to result otherwise.

The speaker believes in antiseptic surgery so long as it forces upon us greater cleanliness, but he is not yet fully satisfied that the micro-organisms which are supposed to be destroyed by antiseptic lotions, etc., are the causes of the disease. They may be simply sequences. The disease may furnish them suitable pabulum upon which to grow, and they develop as a result of the disease. Perhaps, after all, the antiseptics only prevent the furnishing of this *tertium quid* on which the bacilli and micrococci grow and thrive.

This paper drew out considerable discussion with reference to chloroform and ether, the general opinion being that chloroform, under all circumstances and all things being considered, was as safe, if not safer, than ether.

FIRST DAY—AFTERNOON SESSION.

Dr. G. W. Drake, of Chattanooga, read a paper on the triune man, which, from a metaphysical standpoint, was an able disquisition.

It was followed by a contribution entitled "Hypermetropia" by Dr. N. T. Dulaney, of Bristol.

Dr. P. H. McKinnie, of Hickory Valley, read a paper on

PNEUMONITIS AND ITS TREATMENT, in which he offered some good suggestions.

Dr. J. A. Witherspoon, of Columbia, read a paper on Endocarditis and its differential diagnosis, in which he said that the correct diagnosis of heart affections depends upon a thorough understanding of the anatomy and physiology of the organ. He then offered a plea for more thorough examinations, to satisfy ourselves that lesion of the heart exists. We should not be contented with the unscientific diagnosis of "heart disease." It is the sacred duty of the physician to investigate thoroughly and closely before pronouncing the benediction on the activities of a man's life, by saying that he has organic heart disease. If the early manifestations of the disease were recognized early and proper treatment instituted fatal sequelæ might be prevented which it often leaves behind it. Endocarditis occurs in three varieties, viz., exudative, ulcerative, and interstitial. The essayist would confine his remarks to exudative endocarditis which occurs usually in those diseases in which there is marked dyscrasia of the blood, where that fluid is altered in its histological proportions or physiological elements, and especially in acute articular rheumatism, for it occurs in about 33 per centum of such cases. It may occur in any diseases in which there is a morbid state of the blood, such as the exanthemata, essential fevers, diphtheria, nephritis, pyæmia, etc. It may occur, as a primary disease, from exposure to cold, violent efforts, blows or other injuries to the chest, and it sometimes occurs without any discoverable cause. Its most frequent site is upon and around the valves, hence the liability to the production of valvular troubles, and it is generally found upon the left side of the heart. Its morbid anatomy explains many of its physical signs, which the speaker described.

Endocarditis is usually associated with signs of acute pericarditis, swollen and painful articulations, and the acid sweats of rheumatism. The differential diagnosis between endocarditis and pericarditis is not difficult were they not so often associated together. In pericarditis we have no murmur, but a to and fro friction sound ; the impulse of the heart is more feeble and distant, and in the later stage, marked enlargement of percussion dulness ; the sounds of the heart are very feeble and muffled in pericarditis, whereas they are even more distinct—except at the site of murmur—and normal in endocarditis.

The only other disease for which endocarditis may be mistaken, is aortitis, especially if the inflammation is in the first part of the aorta. The diagnosis between them is said to be very often impossible to make, the only distinguishing features being the increase of pain which extends along the trunk of the vessel with marked pulsation and a blowing murmur which is transmitted in the direction of the blood current, and a diffused sensitiveness of the arterial system.

There are two complications of endocarditis which cause entire change of symptoms. One is, that the fibrin, which deposits upon the inflamed endocardium, may be so large as to form a clot, and if this clot is large enough, then symptoms of obstructed circulation to a most alarming extent manifest themselves by a condition of collapse ; the skin becomes cold and cyanotic, the pulse thready and weak, and the heart's action exceedingly irregular, with great struggling for breath. The murmur becomes indistinct, and cardiac dulness is slightly increased ; fainting spells alternate with nausea, vomiting, often delirium, and death comes on in a very short time and closes the scene of most terrible suffering. Then, too, we may have a small clot of fibrin or vegetation from the valves washed into the circulation and carried to different parts, when the well known signs of emboli will appear.

Ulcerative endocarditis is usually septic in its origin and is of rare occurrence. This fatal type of the disease is manifested by great prostration, with marked typhoid symptoms, irregular chills, profuse sweats, extremely rapid pulse, dry red tongue, muttering delirium, followed by vomiting, diarrhœa, deep jaundice and stupor ; marked tenderness found over liver and spleen by palpation; great dyspnœa with Cheyne-Stokes respiration and a scanty, highly colored, albuminous urine.

FIRST DAY—EVENING SESSION.

Dr. Duncan Eve, of Nashville, delivered the Presidential Address. He selected for his subject "Recent Progress in our Profession."

He said the valor, the prudence, the wisdom, the patient industry, the zeal, the accurate observation, the critical examination, the continuous contemplation, the superior learning and the great ability engaged in the advancement and development of this department of our science and art (surgery) have been crowned with happiest results. What heretofore would have startled now becomes commonplace. What once defied the utmost powers of the imagination to conceive now becomes the real occurrence of every-day life.

Surgery has taken old and familiar agencies, and found in them either a new value or, discovering their worthlessness, has used and applied others in their stead. It has invented new instruments and appliances, and found new and better uses for those heretofore known. It is continually enlarging its province and ascertaining and defining its boundaries.

Dr. Bayard Holmes, of Chicago, read a paper on "Secondary Mixed Infection in Scarlet Fever," in which he said that no one doubts to-day that scarlet fever is due to an obligate parasite, and the fact that this parasite has not been cultivated on artificial media and studied is due to the closeness of this parasiticism. It has become so perfectly adapted to the human host that it can exist in no other, and on no artificial media. It must be studied in the same manner as the plasmodium malariæ.

Pirogoff noticed that cases of amputation and other major surgical operations did better in filthy cottages than in large and apparently clean hospitals. In England the cottage system of hospitals was found to result in a smaller death rate than obtained in the large hospitals. Puerperal fever, before antiseptic measures had been applied in obstetrics, was less frequent and destructive in country practice than in great hospitals.

The essayist said that everything points to the fact that scarlet fever is a septicæmia self-limited in its nature and finding its atrium of invasion in the pharynx and probably in the tonsil, and manifested by all those symptoms which characterize septicæmias of other kinds.

One of the most frequent symptoms of an unfavorable course of scarlet fever is the enlargement of the cervical lymph-glands. These glands are found crowded with chain cocci, and the same condition may be traced to the tonsil and the walls of the pharynx, the connective tissue of which is found crowded with the same streptococci.

It may be well to consider how this infection is determined. The primary lesion of scarlet fever is without doubt at the seat of invasion, the pharynx. Here is determined a point of least resistance through which the secondary infection which has been demonstrated by all observers finds its atrium.

Dr. W. F. Glenn, of Nashville, read a paper on "Phenique Compounds in Germ Diseases."

He said by the experiments of Pasteur and Tyndall and others, the fact that many, if not all diseases, are due to the presence and active development of living germs in the system has been thoroughly established.

The development of germs in the living system is as properly called fermentation, as is a similar process in the winevat in the formation of wine. If any agency be employed which directly lessens the fermentative process, just in the same proportion is the temperature lowered, and *vice versa*. So we find in the human being, in all zymotic diseases, when the fermentitial microbe is introduced into the blood, there finding its natural element, its development or multiplication begins and the immediate elevation of temperature takes place. Just in proportion to the activity of this multiplication of disease cells, their rapidity of development, so is the fever higher and the symptoms graver.

If we can introduce into the blood or apply to a given part any substance that will destroy the life of these disease germs, we will have reached a perfection of scientific treatment.

From the experiments of Dr. Calvert, it is known that the salts of quinine, in solution, will destroy all vegetable fermentation, the salts of mercury, in solution, all animal fermentation, and a solution of pure carbolic (phenique) acid both.

Can we put a sufficient amount of carbolic acid in the blood, to arrest and prevent the growth of disease germs with safety to our patient? Yes. The accidents heretofore attributed to the administration of carbolic acid have been due to its impurity, more than to the quantity given. If absolutely pure, it is a safe and efficient remedy; if in the least degree impure, it is a very dangerous one.

Dr. Glenn has used Dr. Declat's preparations of phenique, both internally and externally, with gratifying results for the last ten years. During this time he has employed phenique acid as his chief remedy in all cases of malarial, typhoid, and scarlet fevers, diphtheria, erysipelas, blood poison, as a local application to all wounds, whether the result of accident or surgical operation, and has found the result so satisfactory that there is little left to be desired.

In May, 1886, we removed a scirrhus breast, in the private department of the Nashville city hospital, the incision being ten inches in length, extending into the axilla. The wound was dressed with a one to four per cent. solution of glyco-phenique, with the result of no suppuration, no unpleasant odor, and perfect healing in ten days.

Since he has been following this plan of treatment, in typhoid fever, for example, he has never had a diarrhœa to occur in any of his patients; never a hemorrhage from the bowel; never a serious tympanites; never a death. He believed that, with phenique acid and antifebrin as an aid, physicians possessed the treatment, *par excellence*, of all zymotic diseases.

Dr. B. P. Key, of Chattanooga, read a paper entitled "Report of Seven Cases of Ovariotomy in the Last Ten Years," in which he records but one death.

CASE I was a laparotomy for acute peritonitis. Mrs. H., aged 36, was seen by him for the first time on January 20, 1889. About two weeks before this time she was examined by a physician who introduced a sound into the uterus, and from that time on she had been very ill. . . . Patient vomited everything she took into the stomach; breath was very offensive; abdomen slightly tympanitic and very tender; abdominal muscles rigid; bowels constipated; temperature 103½; respiration 24; pulse 120.

He decided at once to open the abdomen. The surroundings of the patient for an operation of so grave a character were very bad. She lived in a little one room board house; any amount of cobwebs and dirt overhead. With all these difficulties staring him in the face he felt a little disinclined to operate, but finally decided to do the operation if the patient was willing to take the chances. She expressed her desire to have an operation done. After using all the antiseptic precautions possible to be carried out in such a dirty place, he laid open the abdomen by an incision of about two and one-half inches, cleansed the abdominal cavity with hot water, closed up the abdominal incision with six cat-gut sutures. Twenty-four hours after the operation the temperature of the body fell to 101, and in forty-eight hours it fell to 99. From this time on patient had an uninterrupted recovery. On the eighth day he removed the dressings and found perfect union of the parts. Recovery.

Case V was the fatal one. Mrs. H., aged 35 ; married about ten years; mother of three children. During the last three years she had suffered intensely from dysmenorrhœa; general health greatly impaired, and the best part of the time was spent in bed; she had constant pains in the right and left ovarian regions. Operation October 2, 1889. At the time of the operation the temperature was 102½, respiration 30, pulse 120. He stated to the patient's family that there was no chance to save her life only by doing a laparotomy. She readily consented to undergo the operation. For the first twenty-four hours after the operation she appeared to rally and continued to improve until the fourth day had passed, when the heart commenced beating very rapidly and she at once became very restlesss. . . . The ovaries were enlarged and contained pus. Patient died on the fifth day.

Dr. F. T. Smith, of Chattanooga, read a paper on "Blindness," which was followed by a contribution on "Eye Symptoms in General Medicine."

Dr. J. S. Cain, of Nashville, read a paper on the "Use and Abuse of Antipyretics."

Antipyretics may be defined as therapeutic means which have for their object the prevention of the accumulation or storage of animal heat within the human economy, above the normal standard of health, which condition is termed pyrexis.

The condition of pyrexis then is the result of two distinct processes, one the increase in heat production, the other a decrease in heat dispersion, either acting singly or together.

When in health or in a physiological state, those two processes exist in such harmony and perfect equipoise, that no matter what may be the circumstances surrounding the individual, the body temperature remains practically at the same standard of 98.5 Fahr., which we term normal temperature.

Certain disease producing agencies gaining admission into the economy through the lungs, stomach or other avenues of invasion, and acting through the nervous system, interfere with or destroy this equalizing process and result in the accumulation of an undue amount of heat in the tissues and blood, and constitute the condition usually termed fever, to which is attached so much importance as a factor in disease. In these views the author is sustained by Auerbach, Traube, Wachsmuth.

The views made prominent by hydrotherapists, that high temperature in fever is the one great condition to be combated, and that it is the cause of the various and grave changes in the brain, heart, kidneys, liver, trophic system, etc., which endanger life, he did not believe was sustained by even the majority of German thinkers, who seem to have almost appropriated the idea of hydrotherapy in fevers. That there is danger from long continued hyperpyretic temperature he had no doubt. But the symptoms of degeneration of the organs above mentioned seem —to use the language of Dr. Von Ziemssen—" not to be the effects of high temperature, but coincident effects of the cause of the fever, and the cause is infection."

Dr. Cain is convinced that pyrexia is not only most often an important factor in disease which does not require any special treatment, but that sometimes and in certain characters of fever, it is one of nature's efforts at repair which should be encouraged; and to combat or antagonize it, in the sense of arresting its generation, is to do the thing of which physicians are sometimes guilty, trying to defeat the very conservative and reparative processes, by which nature is struggling to preserve the life of the patient against the spoliations of the doctor.

In the theory that tissue waste material does contribute largely to the process of oxidation, and by its excessive or diminished production does modify heat elaboration, he is sustained by the best authorities, among whom he mentioned Dr. N. S. Davis, Liebermeister, Winderlisch.

Dr. Sentator has given a detailed exposition of his opinions upon the febrile process, as derived mainly from exact experiments upon metamorphosis, and amongst his conclusions occurs the following language : " Rise of temperature is only one symptom and does not constitute the essence of fever. Its explanation has not yet furnished any theory of fever, because underneath this symptom very different morbid processes are combined, and because there are in fever other important processes, independent of the elevation of temperature." Amongst these Dr. Sentator considers "chiefly the special tissue changes," and further considers that "tissue change is an important process in the elevation of temperature," and that oxidation is the demonstrable, though probably not the sole source of heat."

With regard to antipyretic remedies, those are very properly divided by Dr. Brunton into two main classes, those which lessen the production of heat, and those which increase its loss. The first class is subdivided into those remedies which lessen production by arresting tissue metamorphosis. At the head of this class stands quinia ; also those which lessen oxidation by controlling the circulation and cutting off the oxygen supply which is furnished alone by the blood. These are usually nerve and heart sedatives, and means which retard the active flow of blood. They are again divided into general and local remedies. Under the head of the former class (leaving out venesection now no longer practised) may be mentioned antimony, ipecac, aconite, veratrum viride, gelsemium, and the new and just now much used products of coal tar, antipyrine, antifebrin or acetanilid, phenacetin and others. The therapeutic action of these latter remedies in controlling temperature is not well understood, but when we consider their direct depressing effects upon the medullary and spinal centers, and observe the marked cyanosis nearly always attending their free administration, the conclusion is almost irresistible that they retard oxidation, and by their powerful sedation lessen the supply of oxygen to the tissues, like the other agents mentioned, and lower temperature by arresting combustion.

It is but justice to these latter remedies to say that they also possess other and valuable therapeutic properties ; their marked analgesic powers, and their antiferment and antiseptic influences, as claimed by some, render them very potent agents for good in certain cases where indicated, and they fill a therapeutic want long felt by physicians, but they are as equally potent for evil when improperly or untimely employed.

The following papers were also read :

The Abuse of Quinine, by W. C. Bilbro, M. D., of Murfreesboro.

Suppurative Inflammation of the Middle Ear, by J. C. Sinclair, M. D., of Nashville.

Incontinence of Urine in Children, by A. J. Swaney, M. D., of Gallatin.

Fractured Thigh ; Easy Method of Treatment without Long Splint or Pulley, by J. W. Davis, M. D., of Smyrna.

Hysterectomy for the Removal of a Large Supperitoneal Fibroid, by W. B. Wilson, M. D., of Pulaski.

Fracture of the Lower End of the Radius with Complications, by J. W. Brandon, M. D., of Stribling.

Foreign Body in a Child's Stomach, by S. W. Sanford, M. D., of Henning.

The Importance of Early Treatment in Middle Ear Disease, by N. C. Steels, M. D., of Chattanooga.

Epulia, with Report of Five Cases, by W. B. Rogers, M. D., of Memphis.

Officers for 1891:

President—Dr. G. A. Baxter, of Chattanooga.

Vice-Presidents—Drs. S. W. Sanford, of Henning ; Dr. W. G. Ewing, of Nashville ; N. T. Dulaney, M. D., of Bristol.

Secretary—Dr. D. E. Nelson, of Chattanooga.

Treasurer—Dr. J. P. C. Walker, of Dyersburg.

Place of next meeting, Nashville, Tennessee, second Tuesday in April, 1891.

