# Correction

**Published:** 1890-06

**Authors:** 


CORRECTION.—On page 165 of May issue of the JOURNAL, the title of Dr. Key's paper appeared as "Report of Seven Cases of Ovariotomy in the Last Ten Years," while it should have been "Laparotomy.—Report of Seven Cases in Last Year."

